# Th17 cells and inflammation in neurological disorders: Possible mechanisms of action

**DOI:** 10.3389/fimmu.2022.932152

**Published:** 2022-07-22

**Authors:** Yajun Shi, Bin Wei, Lingjun Li, Bin Wang, Miao Sun

**Affiliations:** Institute for Fetology, The First Affiliated Hospital of Soochow University, Suzhou City, China

**Keywords:** Th17 cells, neurological disorders, IL-17A, neuroinflammation, immune system, microglia

## Abstract

Neurological disorders (NDs) are one of the leading causes of global death. A sustained neuroinflammatory response has been reported to be associated with the pathogenesis of multiple NDs, including Parkinson’s disease (PD), multiple sclerosis (MS), Alzheimer’s disease (AD), amyotrophic lateral sclerosis (ALS), and major depressive disorder (MDD). Accumulating evidence shows that the recruitment of abundant lymphocytes in the central nervous system may contribute to promoting the development and progress of inflammation in neurological disorders. As one subset of T lymphocytes, CD4^+^ T cells have a critical impact on the inflammation of neurological disorders. T helper (Th) 17 is one of the most studied CD4^+^ Th subpopulations that produces cytokines (e.g., IL-17A, IL-23, IL-21, IL-6, and IFN-γ), leading to the abnormal neuroinflammatory response including the excessive activation of microglia and the recruitment of other immune cell types. All these factors are involved in several neurological disorders. However, the possible mechanisms of Th17 cells and their associated cytokines in the immunopathology of the abovementioned neurological disorders have not been clarified completely. This review will summarize the mechanisms by which encephalitogenic inflammatory Th17 cells and their related cytokines strongly contribute to chronic neuroinflammation, thus perpetuating neurodegenerative processes in NDs. Finally, the potential therapeutic prospects of Th17 cells and their cytokines in NDs will also be discussed.

## 1 Introduction

Neurological disorders (NDs) are highly prevalent and have become the second most frequent cause of mortality, with approximately 276 million cases worldwide to date (1). With the global population aging, NDs are recognized as a significant public health challenge today (1, 2). At the same time, the causes and mechanisms behind most NDs are still vague. Though both genetic and environmental factors had been suggested in the etiology of NDs (3, 4), increasing evidence indicates that neuroinflammation is one of the defining characteristics of NDs.

As a pivotal part of the central nervous system (CNS) innate immunity, neuroinflammation initially restrains infection and eliminates pathogens, cell debris, and aggregated or misfolded proteins in a generic manner. While in chronic NDs, neuroinflammation becomes continuous and is harmful to neuronal cells. Increasing evidence has suggested that neuroinflammation contributes to various NDs, including Parkinson’s disease (PD), amyotrophic lateral sclerosis (ALS), multiple sclerosis (MS), Alzheimer’s disease (AD), and major depressive disorder (MDD) (5–7). One of the common traits of these NDs is their neuropathological interactions with the microglia (the resident macrophages in the brain) and astroglia, which trigger an innate immunological response, contributing to the disease’s severity and course (8). The pathogenesis of these diseases was not only restricted to the immunological mechanisms in the brain but also had a strong interaction with the systemic immune system.

Additionally, various pieces of evidence demonstrate that the communication between the CNS and systemic immune systems is possible through immune cells and cytokines breaking the integrity of the blood–brain barrier (BBB) (9, 10). It has been demonstrated that blood-borne immune cells (e.g., CD4^+^ T cells, Th cells) are highly neurotoxic and represent an additional crucial mediator of neuroinflammation. Systemic inflammation, induced by oxidative stress damage, environmental stressors, gut microbiota imbalance, etc., may trigger microglial activation and ultimately contribute to the development of neurological disease processes, according to abundant evidence (8, 11, 12).

Among the critical neuroinflammatory cells in NDs, Th cells, especially Th17 cells, have already been known to be involved. At the same time, various sources of evidence have indicated that Th17 cell levels in both serum, cerebrospinal fluid (CSF), and brain were elevated in the laboratory model animals of NDs (13–15). However, the accurate role of neuroinflammation mediated by Th17 cell and their cytokines in the etiology of the abovementioned NDs is still unclear. With a focus on the roles of Th17 cells and their cytokines in NDs, we have attempted to unravel the precise mechanism of neuroinflammatory disorder in this study. In addition, we provide an overview of the most recent developments in targeting Th17 cells and their cytokines, as well as their prospective clinical applications.

## 2 Th17 cells

### 2.1 Definition and differentiation of Th 17 cells

After encountering specific pathogens, the innate immune system will promote the development of naive CD4^+^ T cells into effector Th cells, which are fundamental regulators of adaptive immunity. Different subtypes of CD4^+^ Th cells are distinguished according to cytokines, transcription factors, and effector immune regulatory functions. Initially, activated CD4^+^ Th cells were frequently differentiated into one of two fates: Th1 or Th2 cells. Th1 cells, generating interleukin (IL)-2, interferon-gamma (IFN-γ), and tumor necrosis factor-alpha (TNF-α), mainly take part in delayed-type hypersensitivity reactions and cell-mediated immune responses, such as autoimmune disorders. Th2 cells, renowned for their generation of the cytokine IL-4, mediate host defense against helminths and link with the pathogenesis of allergic diseases. In 2005, a newly identified subtype of CD4^+^ Th cells, so-called Th17 cells, was discovered that represent a diverse population, which underwent differentiation in the presence of transforming growth factor-beta (TGF-β) and IL-6 (16, 17). Now, it was recognized that Th17 cells might be more significant than Th1 cells in the development of some models of autoimmune illness, including PD, AD, MS, ALS, and MDD (5–7).

Th17 cells perform crucial roles in pro-inflammatory properties, inflammation, and essential tasks to defend the host against extracellular bacterial and fungal infections. Th17 cells are defined as CD4^+^ Th lymphocytes that secrete large amounts of IL-17A and express the transcription factor retinoic acid-related orphan receptor gamma-T (RORγt), which possibly acts as a molecular determinant for their polarization (18, 19). Th17 cells have two most distinct characteristics: strong plasticity and prominent capability to boost other immune cells (e.g., Th1 cells, neutrophils, and immunosuppressive head box P3 Treg cells).

**Figure 1:**
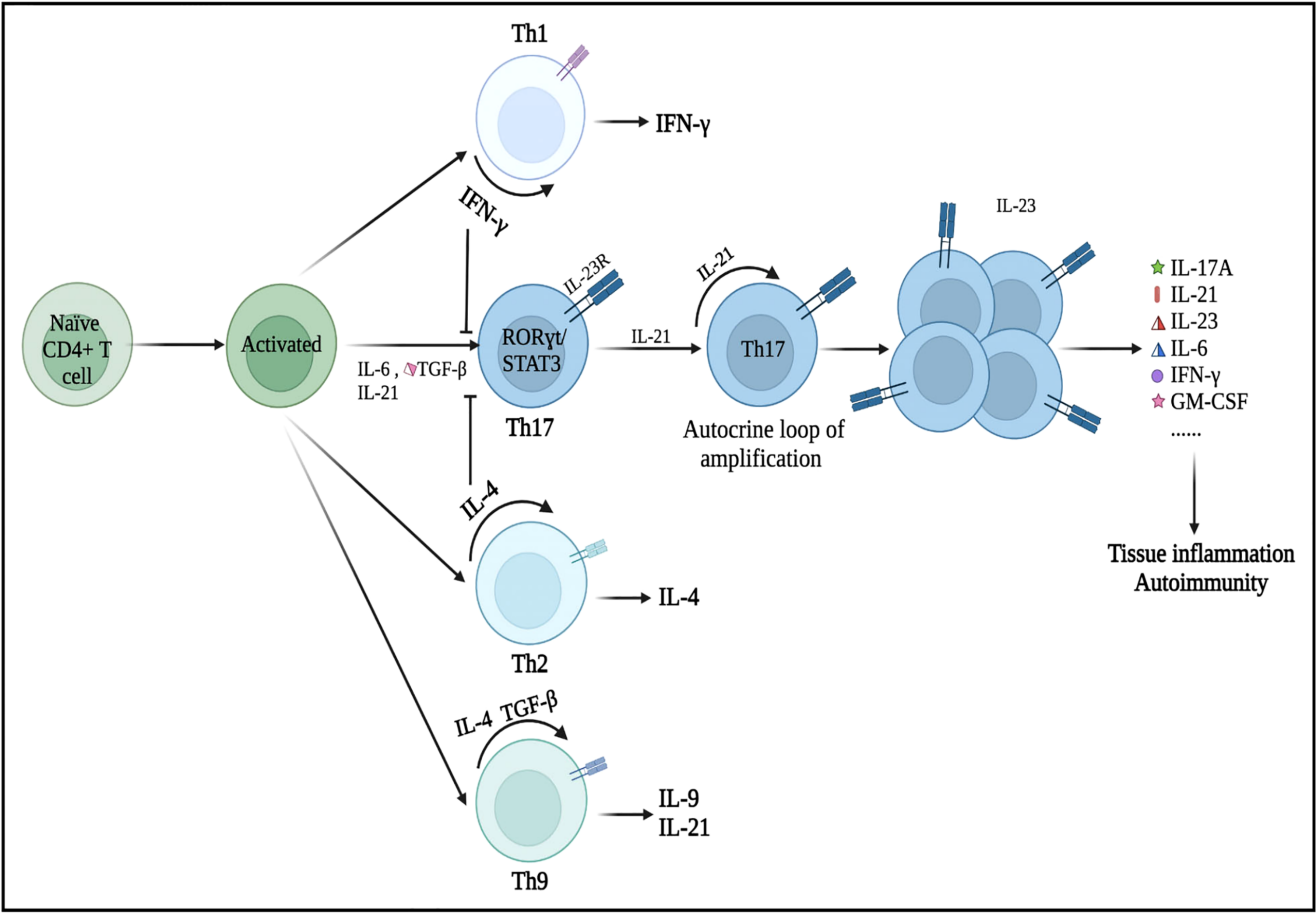
Differentiation of Th17 cells and its secretion of inflammatory cytokines. Under the microenvironment of pro-inflammatory cytokines (IL-6, IL-21, and/or TGF-β), naive CD4^+^ T precursors can be induced to differentiate into Th17 cells, which have crucial roles in the immunopathogenesis of neurological disorders. Nonetheless, IFN-γ (a Th1 cell-related cytokine) and IL-4 (a Th2 cell-related cytokine) can inhibit the differentiation of Th17 cells from naive CD4^+^ T cells as long as Th17 cells have not been fully developed. These primary differentiated Th17 cells will express specific transcription factors, such as RORγt and STAT3. IL-21 is produced by Th17 cells in an autocrine manner and other cells then promote Th17 cell proliferation and differentiation (expansion stage). IL-23 promotes the terminal differentiation, development, and survival of expanded Th17 cells in their final stable state. IL, interleukin; TGF-β, transforming growth factor-β; IFN-γ, interferon-γ; STAT, signal transducer and activator of transcription.

Initially, the differentiation of Th17 cells was shown to be induced by the combination of IL-6 and TGF-β (20). Subsequently, Korn and colleagues found that IL-21, which was induced by signal transducer and activator of transcription 3 (STAT3)/IL-6 signaling, could affect Th17 cell development and response amplification (21). IL-21 acts in a loop of autocrine amplification to culminate in the differentiation and proliferation of Th17 cells and the production of the IL-23 receptor (IL-23R). IL-23 plays a key role in the late growth and maturation of Th17 cells by activating its receptor IL-23R *via* IL-6 and/or IL-21 (**Figure 1**) (22). Additionally, Th17 cells differentiated in the IL-1^+^/IL-23^+^/TGF-β^−^ environment show greater encephalitogenic activity after adoptive transfer, which highlights the importance of IL-23 and IL-1 in the differentiation and pathogenicity of Th17 cells (23). Nonetheless, IFN-γ (Th1 cytokine) and IL-4 (Th2 cytokine) can inhibit the differentiation of Th17 cells from naive CD4^+^ Th cells as long as the Th17 cells have not been fully developed (24, 25).

RORγt, which was encoded by the *RORC* gene and generally expressed in Th17 cells, is the major regulator of the Th17 cell differentiation. *Rorc* knockout mice cannot produce Th17 cells (26). Meanwhile, overexpression of RORγt, triggered by the activity of IL-6 and TGF-β, can lead to Th1- and Th2-independent differentiation of naive CD4^+^ Th cell progenitors into the Th17 subtype (27, 28). Moreover, RORγt may cooperate with other transcription factors during the differentiation of Th17 cells (29). When activated by IL-6, IL-21, or IL-23, STAT3 is the upregulator of RORγt in response to TGF-β and IL-6. STAT3 can bind to plenty of Th17-related loci and is essential for the induction of the Th17 transcriptional program (30). In the absence of STAT3, overexpression of RORγt cannot restore Th17 cell development (31). Numerous essential transcription factors, such as STAT5, granulocyte monocyte-colony stimulating factor (GM-CSF), aryl hydrocarbon receptor (Ahr), activating transcription factor-like runt-related transcription factor-1 (RUNX1), transcriptional coactivator with postsynaptic density 65-discs large-zonal occluder 1-binding motif (TAZ), and interferon regulatory factor 4 (IRF4), regulate Th17 cell differentiation (29, 32, 33). Th17 cells are regarded as strongly pathogenic because they drive autoinflammation by producing a unique set of cytokines (e.g., IL-17A, IL-21, IL-23, IL-6, IFN-γ, and GM-CSF) (**Figure 1**).

It has been shown that IL-17A, mainly produced by Th17 cells, can induce cytokine secretion and has functions in immune inflammation (34). Numerous studies signify that IL-17A, stimulating glial cells and enhancing neuroinflammatory responses, performs a pathogenic role in the CNS (35, 36). IL-17 signaling also induces the activation of NF-κB cascade response to control the corresponding physiological function (37).

### 2.2 Environmental factors that affect the pathogenic potential of Th17 cells

Increasing evidence indicates that multiple environmental factors will contribute to the development of NDs. It is not difficult to think that different host environmental factors may also accelerate the pathogenic potential of Th17 cells and that immune responses may promote autoimmunity, therefore causing NDs.

#### 2.2.1 Peripheral inflammation

Numerous studies indicated that Th17 cells and their cytokines would compromise the integrity of the BBB. Thus, the migration of immune cells from the periphery into the central nervous system has become one of the major contributors to numerous NDs (38, 39). For instance, *Porphyromonas gingivalis* stimulates the production of Th17-supportive cytokines (IL-1, IL-6, and IL-23), which may permeate the BBB to endotoxin and expose the brain cells to endogenous and exogenous inflammatory mediators (40). Th17 cells and their related cytokines are also associated with abnormally aggregated plaques of the protein amyloid-beta (Aβ) in neurons (41). In a mouse model of PD, Vitola and colleagues demonstrated for the first time that peripheral injection of lipopolysaccharide could trigger a long-lasting increase in the pro-inflammatory cytokine TNF-α, induce neuroinflammation, influence the action of α-synuclein (αSyn) oligomers, and potentiate the detrimental effects (42). Moreover, Th17 cells, immune cells, and macrophages are abundant in the peripheral blood of MS patients, which would infiltrate the CNS and collaborate to cause tissue damage (43). The entry of peripheral immune cells from the periphery into the CNS can also be viewed as a precursor to the breakdown of the BBB, thereby heightening brain inflammatory responses (44). Rodent models suggested that vagal stimulation can increase the levels of brain cytokines in response to prolonged peripheral inflammation (45). Interestingly, the response to acute peripheral inflammation of the microglia is not associated with long-term neuronal damage. Only in chronic and slowly progressive diseases (such as AD, MDD, and PD) are the microglia activated by peripheral (cytokines) and/or brain [Aβ, oxidative stress (OxS), etc.] pro-inflammatory stimulus to prime for further neurotoxicity (8). As evidence, ND patients and animal models with persistent inflammatory neurodegeneration exhibited an acceleration of the neurodegeneration process in response to peripheral inflammatory stimulus (46, 47).

#### 2.2.2 Enhanced oxidative stress

Indeed, one of the mechanisms underlying the pathogenesis of NDs is the vicious cycle between OxS and neuroinflammation, which is reflected in the brain and peripheral immune system (8). As elegantly discussed by Cobley and colleagues, the brain is highly sensitive to oxidative damage for a variety of reasons, including hypoxia/ischemia, glutamate, mitochondrial dysfunction, endogenous neurotransmitter metabolism (e.g., dopamine), and abnormal activation of microglia (48). Under the condition of chronic oxidative stress, mitochondrial hyperpolarization, nitric oxide production, and Ca^2+^ influx were enhanced, and the overexpression of the mechanistic target of rapamycin complex 1 (mTORC1) was facilitated (49). Furthermore, the activity of mTORC1 promotes the expansion of Th17 pro-inflammatory lymphocytes from CD4^+^ T cells, which constitute a critical CNS mechanism of immune regulation. After activation, CD4^+^ T cells can easily cross the BBB. As a result of the presence of IL-23, CD4^+^ T cells differentiate into Th17 cells when they reach the damaged site. In intestinal immunity, Th17 cells can contribute to neuroinflammation and neurodegeneration by activating the apoptotic Fas/FasL pathway (50). OxS has been ascribed as a major contributor to the pathogenesis and clinical progression of NDs (11, 50).

#### 2.2.3 The gut–brain axis

Considerable evidence suggests that the gut microbiota is essential for normal host metabolism and physiological function, influencing not only the immune system but also the nervous system (51, 52). The gut microbiota is also related to brain development and cognitive function. The gut microbiota affects the gut–brain axis *via* direct and indirect (*via* systemic circulation) pathways, including immune (cytokines), endocrine (cortisol), and neural (enteric nervous system) pathways (53). Diet-induced alterations to the gut microbiome can result in a compromised lamina propria, where Th17 cells are the predominant Th cells (26). Recently, Th17 cells, inducing the production of IL-17A and IL-22, have been shown to play distinct roles during fungal infection and colonization (54, 55). The importance of gut microbiota, particularly segmented filamentous bacteria (SFB), in the generation of mucosal Th17 cells was first demonstrated by Littman and colleagues (56). Surprisingly, colonization of the gut with SFB alone can induce the differentiation of Th17 cells in the gut and CNS, thereby accelerating the progression of NDs (57). Chen et al. have proven that the major metabolite of clostridia species, short-chain fatty acid butyrate, is sufficient to restore BBB dysfunction in patients with AD (58). The disruption of the gut microbiota stimulates the activation of astrocytes and microglia, which in turn influence a variety of neuropsychological processes (e.g., neuronal development, BBB integrity, CNS immune system activation) (59). Various environmental factors modulate the immune system’s response, strongly accelerating the pathogenic potential of Th17 cells. Indeed, alterations of Th17 cells in the gut microbiota have been documented in several diseases such as AD (60), PD (61), and MS (62). Under high-salt conditions, a highly pathogenic Th17 cell population activated by the p38/MAPK pathway has been observed (63). Furthermore, the mice on a high-salt diet developed cognitive impairment due to cerebral endothelial dysfunction (64), which was associated with an increase in Th17 polarization and IL-17A plasma level in the small intestine. Moreover, hypoxia promoted Th17 differentiation by activating the hypoxia-inducible factor 1 (HIF-1), a critical metabolic sensor that directly regulates the expression of RORγt and IL-17A at the transcriptional level (65). The gut–brain axis appears to be a potent druggable target for the immunotherapy of IL-17A in NDs.

## 3 Th17 cells and its cytokines in neurological disorders: Possible mechanisms of action

### 3.1 Th17 cells in Parkinson’s disease

**Figure 2:**
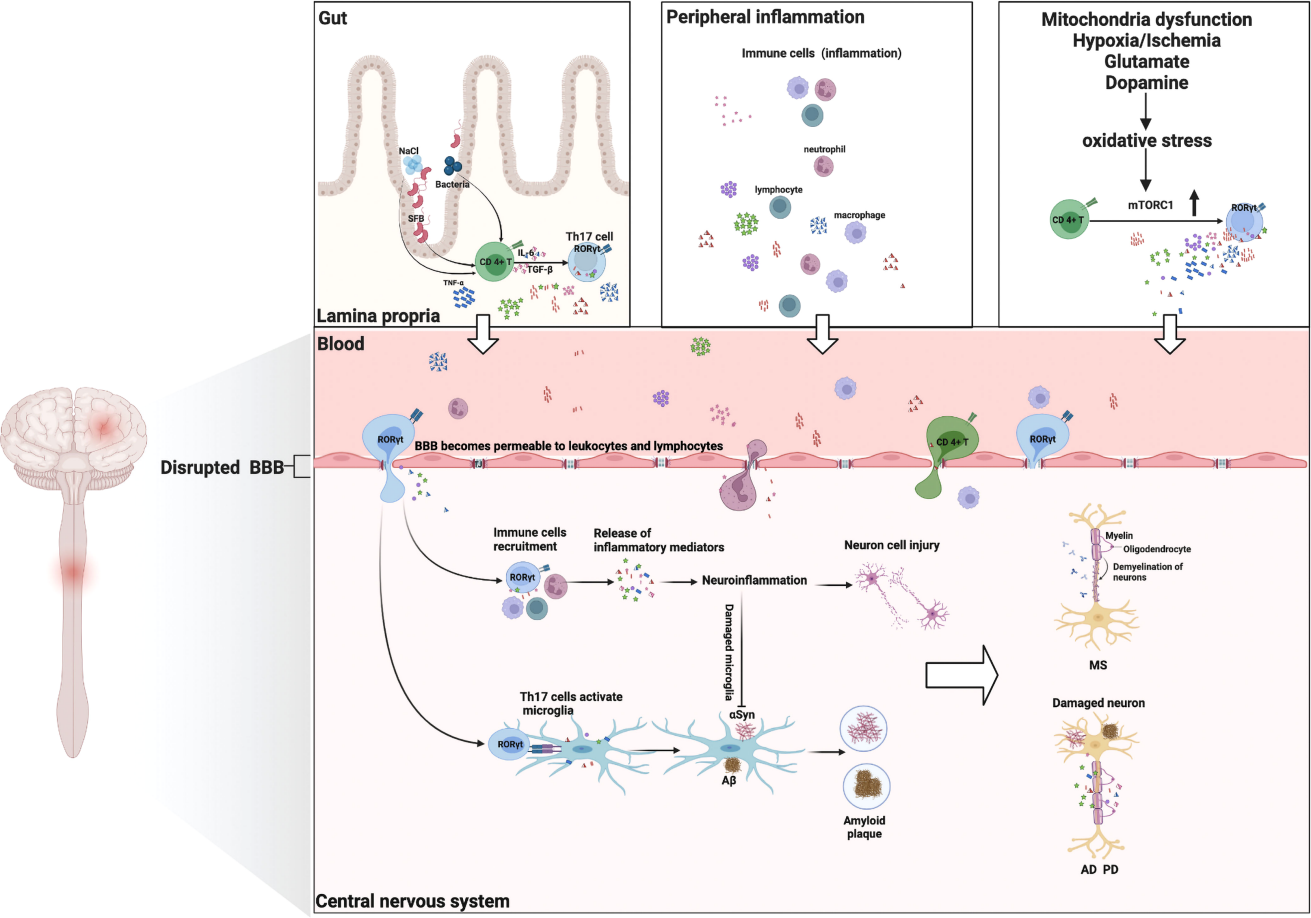
Th17 cells and their cytokines in neurological disorders: possible mechanisms of action. Multiple environmental factors (peripheral inflammation, enhanced oxidative stress, gut–brain axis) induce a pro-inflammatory microenvironment that modifies the CD4^+^ T-cell phenotype and then differentiates into encephalitogenic Th17 cells, producing inflammatory cytokines (IL-17A, IL-6, IL-21, IFN-γ, GM-CSF, and IL-23). These Th17 cells are capable of entering the CNS. They proliferate and produce cytokines that are conducive to BBB disruption and recruitment of other immune cells (lymphocytes, macrophages, and neutrophil cells) into the CNS, ultimately leading to myelin damage (multiple sclerosis). Th17 cells and their cytokines can cause neuronal damage through direct cytotoxic effects or through recruitment of immune cells and induction of neuroinflammation, resulting in deposition of Aβ fibrils or the aggregation of αSyn (Alzheimer’s disease/Parkinson’s disease). Additionally, Th17 cells can activate the microglia and phagocytose amyloid fibrils, but neuroinflammation will induce microglia damage, thereby exacerbating amyloid β deposition or αSyn aggregation (Alzheimer’s disease/Parkinson’s disease). GM-CSF, granulocyte monocyte-colony stimulating factor; Aβ, amyloid β.

PD, the second most prevalent form of neurodegenerative disease, is characterized by motor symptoms, such as tremors, rigidity, and bradykinesia (66). In PD, there is a progressive degeneration of neurons in various regions of the brain, including dopaminergic neurons in the substantia nigra pars compacta (67). A critical pathological hallmark of PD is the accumulation of αSyn (so-called Lewy bodies). In addition to protein aggregation, inflammatory responses play a crucial role in the etiology and pathogenesis of PD, as evidenced by the elevated expression of IFN-γ, IL-17A, and IL-6 in the brain (68). PD patients also have a disruption of BBB, which allows peripheral immune cells to infiltrate into the brain and potentially influences other mechanistic pathways associated with neurodegeneration, such as oxidative stress and mitochondrial dysfunction potentially (68). Therefore, the enhanced peripheral inflammation may initiate or exacerbate PD pathology. Recently, Sommer et al. provided direct evidence that the neurotoxic effect of Th17 cells, expanded in an autocrine loop, was increased in PD patients (69). This model also revealed a direct contact between Th17 cells and neurons leading to dopaminergic neuronal apoptosis. In a mouse model with a deficiency of IL-17A, motor impairment, dopaminergic neurodegeneration, and BBB disruption were alleviated (70). Furthermore, Gate and colleagues provided the exact mechanism for Th17 cell-mediated dopaminergic neuron death *via* secretion of IL-17A in PD (71). Th17 cells secrete pro-inflammatory cytokines that were associated with the activation of other detrimental inflammatory factors (like TNF-α, IL-1β, and IL-6), which were released from brain microglial cells (the most numerous types of brain cell). Ultimately, Th17 cells and inflammatory factors promote inflammatory reactions and neuronal apoptosis (**Figure 2**). On the other hand, abnormally accumulated αSyn becomes an autoimmune antigen that activates microglia into the microglia type 1 subtype, which promotes the differentiation of Th17 subtypes and activates intracellular inflammatory pathways (72). Although the direct effects of Th17 cells on neurons have been described in these studies, additional work is still needed to comprehend the activity of PD-related Th17 neurotoxicity in a more complex intracellular environment to open up novel therapeutic development avenues.

### 3.2 Th17 cells in Alzheimer’s disease

AD is the most frequent form of late-onset dementia and has progressive and irreversible pathogenesis with a complex molecular disease etiology, which affects over 40 million people worldwide (73). AD is a multifactorial neurological disorder, in which the extracellular deposits of amyloid β (Aβ) peptides and fibrillary tangles of intraneuronal hyperphosphorylated tau protein are the major histopathological hallmarks. Aβ is obtained by the frequent and serial activation of the γ-secretase enzymes and β-secretase 1 (BACE1) on a larger precursor protein (APP) (74). Highly insoluble Aβ peptides are considered an important factor in AD pathogenesis. They motivate the microglia to secrete pro-inflammatory cytokines and chemokines to activate the complement pathway (75), thus inducing the cumulation of inflammatory cells into the CNS that leads to neurodegeneration (76). Indeed, Aβ peptides have been shown to enhance the manufacture of reactive oxygen species (ROS) and nitric oxide (NO) by microglia, leading to OxS development and stimulating the inflammation of the Th17/IL-17A axis, and then impair microglia-mediated Aβ phagocytosis and contribute to Aβ accumulation and neuronal damage. Additionally, the primary roles of IL-17A in the pathogenesis of AD are to attract neutrophils and then stimulate their function. Zenaro et al. showed that Aβ aggregates could mediate the recruitment and chemotaxis of neutrophils to produce IL-17A, which is directly toxic to neurons and the BBB and may amplify neutrophils in the CNS, thus contributing to a vicious circle that leads to exacerbating pathology (77). Since neutrophils are the primary targets and crucial sources of IL-17A in the CNS, they may play a significant role in the development of AD pathology by promoting inflammation and neuron autophagy. The presence of extremely high levels of IL-17A, TNF-α, IL-2, and GM-CSF in the brain of a triple transgenic mouse model of AD indicates that neurodegeneration in these mice is related to Th17 polarization. Another study found a notable increase of IL-17A, RORγt, and IL-22 in the serum, hippocampus, and CSF of Aβ-42 peptide-injected rats (78). In addition, Tian et al. indicated a correlation between postoperative cognitive impairment and elevated levels of IL-17A in the hippocampus (79). Recently, a study has reported that the administration of blocking anti-IL-17A antibodies could rescue Aβ-induced cognitive impairment and neuroinflammation (80). These findings provide evidence for the synergistic roles of Th17 cells and their associated cytokines in promoting neuroinflammation and degeneration in AD.

The number of CD4^+^ and CD8^+^ T cells in AD patients’ brain parenchyma and vascular endothelium is significantly higher than normal (81). The activated Th17 cells and their inflammatory cytokines (e.g., IL-17A, IL-21, and IL-23) in the brain may jointly promote AD neuropathology (82). Furthermore, a number of studies found that AD patients had elevated levels of the Th17 transcription factor RORγt in their lymphocytes (83, 84). Recently, other studies have demonstrated the relationship between Th17 cells and early AD (85). Therefore, some scholars proposed that the optimal AD vaccine should inhibit Th17 immune responses to Aβ to prevent neuroinflammation and subsequent neurodegeneration (86).

### 3.3 Th17 cells in multiple sclerosis

MS is a widespread demyelinating disease of the CNS resulting in substantial neurological disability, in which immune cells and related cytokines are involved in the degradation of myelin sheaths (87). Inflammatory processes in these foci led to myelin damage and oligodendrocyte rupture, followed by axonal loss and transient or permanent loss of neurologic functions, resulting in varying degrees of disability (88). Until now, the pathophysiology of MS has not been elucidated clearly. Still, the prevailing view of MS pathogenesis is the breach of immunological tolerance and the active infiltration of myelin antigen-sensitive immune cells into brain tissue through the BBB. Emerging data from clinical and animal studies have revealed that myelin-specific immune cells (such as B cells, Th1 cells, and Th17 cells) are activated in lymph organs of the periphery. These myelin-specific immune cells develop encephalitogenic potential and infiltrate the CNS, where they are reactivated and expanded by the IL-23 and IL-1β (produced by resident microglia and infiltrating inflammatory monocytes) (62, 89). Furthermore, multiple studies have indicated that Th1 and Th17 cells are enhanced in the brain parenchyma in the acute phase of MS patients (90, 91). Th17 cells mainly cause brain damage, but Th1 cells induce spinal cord inflammation. In the attempt to clarify the role of Th1 and Th17 cells in the pathogenesis of MS, Langrish et al. showed that Th17 cells induce more severe experimental autoimmune encephalomyelitis (EAE) than Th1 cells (92). Th17 cells, especially Th1-like Th17 cells, may participate in EAE pathology by producing IFN-γ and IL-17A. Th17 cells result in oligodendrocyte death, axonal degeneration, and neuronal dysfunction (91, 93). Multiple studies in various EAEs, animal models of MS, have demonstrated that Th1-like Th17 cells can across the BBB by stimulating the IL-17A and C-C chemokine receptor 6 (CCK6) and enhance neuroinflammation (87, 94, 95). In the CSF and peripheral blood of patients with MS relapse, Durelli and colleagues detected that a higher IL-17A expression was positively associated with disease activity (96). Mice that lack Th17 and its characteristic cytokines, including IL-23, IL-21, and IL-22, are also at risk of developing EAE (97, 98). Furthermore, Hartlehnert and colleagues identified the induction of the B-cell-supporting meningeal microenvironment by Bcl6 in Th17 cells as a mechanism controlling neuroinflammation (99). Therefore, IL-17A and B cells have a considerable but non-essential function in EAE. Additionally, mice lacking RORγt, a crucial transcription factor for Th17 differentiation, exhibit a delayed onset or mild progression of EAE (100). Unlike other Th17 cytokines, GM-CSF, regulated by IL-23, RORγt, and IL-1β, has an encephalitogenic profile and a non-redundant role in active myeloid cell infiltration leading to sustained neuroinflammation in MS (101). Although the absence of GM-CSF had no effect on Th cells infiltrating into the CNS, it severely inhibited the accumulation of tissue-invading phagocytes, which are the primary drivers of immunopathology and are capable of initiating tissue damage (101). Consequently, Th17 cells and their related factors have a significant role in the pathogenesis of MS.

### 3.4 Th17 cells in ALS

ALS is a progressive neurodegenerative disease, which is characterized by the clustering and accumulation of ubiquitylated, proteinaceous inclusions in the upper and lower motoneurons (MN), resulting in dramatic muscle paralysis, extramotor abnormalities, or death (102). Although the pathophysiological mechanisms of ALS are still unresolved, multiple studies corroborated that the pathological processes of ALS are involved in neuroinflammation, mainly attributed to the activation of CNS innate immune cells, and may precede MN cell death (35, 103). Neuroinflammation in ALS is characterized by the accumulation of massive astrocytes and activated microglia, which increases the production of potentially cytotoxic molecules (such as ROS, inflammatory mediators, and pro-inflammatory cytokines) (104). Interestingly, recent studies provide evidence that Th17 cells and their associated cytokines (e.g., IL-17A, TNF-α, IFN-γ) are enhanced in the CSF, peripheral blood, and serum of ALS patients (35, 104). In clinical studies with ALS, Meng et al. showed that peripheral pro-inflammatory Th17 cell shift was linked to disease severity (13). These findings proved that therapeutic interventions on Th17 cells and/or their cytokines may be a promising treatment strategy in ALS patients.

### 3.5 Th17 cells in MDD

MDD is one of the most commonly recognized mental disorders globally. The number of patients receiving antidepressant treatment is increasing yearly, which is a severe medical, social, and economic problem. According to the Composite International Diagnostic Interview version of the World Mental Health Survey, MDD is a chronic psychiatric disorder with long-term depressive symptoms (e.g., anxiety, loss of pleasure, and low sense of self-worth). With the steadily rising number of young patients with MDD over the past decade, there is an urgent need for more effective therapeutic strategies and the identification of the molecular mechanisms underlying chronic MDD. Although the etiology of MDD has yet to be determined, mounting evidence supports a link between depression and elevated levels of IL-17A, suggesting that inflammation exacerbates depressive symptoms. Indeed, numerous pro-inflammatory cytokines (e.g., IL-1β, IL-23, IFN-γ, and TNF-α) and Th17 cells are elevated in the peripheral blood of depressed patients (105, 106). Alvarez-Mon et al. clearly showed an increased propensity for Th17 differentiation in the circulating population of CD4^+^ T lymphocytes in adult patients with MDD (105). In addition, several studies have also demonstrated that Th17 cells were increased in the brains of the rodent models of MDD (107). Interestingly, studies showed that administration of Th17 cells would result in increased sensitivity to depression in two different mouse models, giving a direct correlation between Th17 cells and depression sensitivity (108, 109). The absence of RORγt and the neutralization of IL-17A both conferred resistance to the induction of learned helplessness in mice (108). Nevertheless, the levels of IL-17A are not always correlated with depression, and there is no evidence indicating that Th17 cells cause neuron damage in MDD. Similar to psoriasis and MS surveys, patients with rheumatoid arthritis who have elevated levels of IL-17A also have an increased risk of depression and anxiety disorders (110, 111). These findings suggest that Th17 cells may not be sufficient to cause depression on their own, but they have additional roles to promote depression. To clarify whether IL-17A is directly involved in neuroimmune interactions, further research is required.

## 4 Prospective and effective therapeutic strategies targeting Th17 cells and their cytokines in neurological disorders

As demonstrated previously, Th17 cells and their cytokines play crucial roles in the pathogenicity of the immune system in neurological diseases. Consequently, they may be prospective therapeutic targets for NDs due to their pathogenic functions. At present, numerous methods target Th17 cells and their cytokines, including anti-GM-CSF antibodies, anti-IL-17A monoclonal antibodies, and RORγt inhibitors (**Table 1**). Although the efficacy of these techniques in the treatment of NDs involving pathogenic Th17 cells has not yet been demonstrated and is still in the developmental stages, strategies that target Th17 and their cytokines remain promising.

**Table 1:** Drugs targeting Th17 cells and their cytokines.

Drug name	Target cytokine	Mechanism of action	Clinical development	Mouse model		References
Secukinumab	IL-17A	Full human monoclonal antibody targeting IL-17A	Rheumatoid arthritis, spondylarthrosis, psoriasis, MS		NA	(112)
Ixekizumab	IL-17A	Humanized anti-IL-17A monoclonal antibody	Psoriasis, rheumatoid arthritis		NA	(113)
Brodalumab	IL-17R	Full human monoclonal antibody targeting the IL-17 receptor	Psoriasis		NA	(114)
Anti-GM-CSF antibody	GM-CSF	Suppresses microglia activation in the brains of C57/BL6 mice	NA		AD, MS	(115–117)
TAK-828F SR1001	RORγt	RORγt inverse agonists	MS		NA	(118, 119)
Ursolic acid, oleanonic acid	RORγt	RORγt inverse agonists	NA		MS	(120, 121)

### 4.1 Targeting the IL-17A/IL-17AR axis

IL-17A is the critical effector cytokine secreted by Th17 cells. Only three monoclonal antibodies of IL-17A and IL-17AR were approved, namely, secukinumab, ixekizumab, and brodalumab. Secukinumab is a fully human monoclonal antibody, which was labeled as first-line therapy for moderate to severe cases of active or stable psoriasis (122). Macaluso and colleagues first demonstrated secukinumab’s clinical efficacy on neurological manifestations in patients with concomitant ankylosing spondylitis (AS) and MS, supporting the fact that IL-17A blockade may become a potential therapeutic target in MS (112). Cortese et al. also suggested that secukinumab was efficacious for treating CNS demyelination in AS patients, but this topic still needs further studies (123). Moreover, the restoration of neuronal cell death produced by Th17 cells by secukinumab in PD patient-generated pluripotent stem cell-derived neurons motivated us to look for additional possible immunotherapies for PD (69). Ixekizumab, another human monoclonal antibody, inhibits the inflammatory response mediated by IL-17A binding to the IL-17A receptor (IL-17AR), whereas brodalumab can specifically block the IL-17 receptor. Extensive clinical studies have shown that ixekizumab and brodalumab are beneficial and used by the FDA in adults with moderate to severe plaque psoriasis (114, 124). Emerging evidence hints that the therapeutic interventions on IL-17A may be a promising treatment strategy in ND patients. To confirm the efficacy of anti-IL-17A/IL-17AR antibodies in the treatment of NDs, additional research is required.

### 4.2 Targeting other Th17 cytokines and Th17 cell development

In addition to IL-17A, Th17 cells release numerous additional cytokines, including GM-CSF, IL-23, and RORγt. GM-CSF was reported to play a pathogenic role in NDs, such as AD, PD, MS, and MDD. Several studies in AD and PD animal models have demonstrated that anti-GM-CSF antibodies would inhibit the activation of the microglia and astrocytes in the CNS, suggesting that it may be a potential therapeutic target (113, 115). In support of this finding, Manczak and colleagues observed that animals injected with anti-GM-CSF antibodies had decreased Aβ deposition and microglia expression (116). Since IL-23 and RORγt are involved in the differentiation and activation of Th17 cells, targeting the IL-23 or RORγt in the treatment of NDs is exciting and an effective approach (117). Currently, several RORγt inverse agonists, including oleanonic acid, ursolic acid, and TAK-828F, have been identified (118–120). For instance, SR1001 could alleviate the severity and delay the onset of EAE in distinct mechanisms, including limiting Th17 cell differentiation and reducing the expression of Th17 cytokines (117).

## 5 Conclusions and future directions

Since the discovery of the link between Th17 cells and the development of neurological disorders, the world of Th17 cells and their cytokines in NDs has experienced a boom of research and discoveries. Th17 cells have been described to play crucial roles in NDs, but more work is needed to clarify the exact mechanisms of their function in the future. This knowledge is essential for determining the importance of Th17 cells and finding prospective therapies for patients with neurological disorders. Existing data and future directions suggest that Th17 cells and their cytokines and Th17-related signaling pathways will be potentially effective therapeutic neuroprotective targets in the treatment of NDs (121).

## Author contributions

YJS and BW wrote the manuscript with support from LJL, BW, and MS. All authors contributed to the article and approved the submitted version.

## Funding

This work was supported by grants from the National Key R&D Program of China (2019YFA0802600) and the National Natural Science Foundation of China (81974244 and 81570960).

## Conflict of interest

The authors declare that the research was conducted in the absence of any commercial or financial relationships that could be construed as a potential conflict of interest.

## Publisher’s note

All claims expressed in this article are solely those of the authors and do not necessarily represent those of their affiliated organizations, or those of the publisher, the editors and the reviewers. Any product that may be evaluated in this article, or claim that may be made by its manufacturer, is not guaranteed or endorsed by the publisher.
